# A Mycoses Study Group International Prospective Study of Phaeohyphomycosis: An Analysis of 99 Proven/Probable Cases

**DOI:** 10.1093/ofid/ofx200

**Published:** 2017-09-26

**Authors:** Sanjay G Revankar, John W Baddley, Sharon C -A Chen, Carol A Kauffman, Monica Slavin, Jose A Vazquez, Carlos Seas, Michele I Morris, M Hong Nguyen, Shmuel Shoham, George R Thompson, Barbara D Alexander, Jacques Simkins, Luis Ostrosky-Zeichner, Kathleen Mullane, George Alangaden, David R Andes, Oliver A Cornely, Kerstin Wahlers, Shawn R Lockhart, Peter G Pappas

**Affiliations:** 1 Division of Infectious Diseases, Wayne State University, Detroit, Michigan; 2 Division of Infectious Diseases, University of Alabama at Birmingham, Birmingham, Alabama; 3 Birmingham VA Medical Center, Birmingham, Alabama; 4 Centre for Infectious Diseases and Microbiology, Westmead Hospital, and the University of Sydney, Westmead, Australia; 5 Division of Infectious Diseases, University of Michigan Medical School and VA Ann Arbor Healthcare System, Ann Arbor, Michigan; 6 Victorian Infectious Diseases Service, Melbourne Health, Parkville, Australia; 7 Division of Infectious Diseases, Georgia Regents University, Augusta, Georgia; 8 Instituto de Medicina Tropical Alexander von Humboldt, Universidad Peruana Cayetano Heredia, Lima, Peru; 9 Division of Infectious Diseases, University of Miami, Miami, Florida; 10 Division of Infectious Diseases, University of Pittsburgh, Pittsburgh, Pennsylvania; 11 Division of Infectious Diseases, Johns Hopkins University, Baltimore, Maryland; 12 Division of Infectious Diseases, University of California at Davis, Davis, California; 13 Division of Infectious Diseases, Duke University Medical Center, Durham, North Carolina; 14 Division of Infectious Diseases, University of Texas Health Science Center, Houston, Texas; 15 Division of Infectious Diseases, University of Chicago, Chicago, Illinois; 16 Division of Infectious Diseases, Henry Ford Health System, Detroit, Michigan; 17 Division of Infectious Diseases, University of Wisconsin, Madison, Wisconsin; 18 Cologne Excellence Cluster on Cellular Stress Responses in Aging-Associated Diseases (CECAD), and Department I of Internal Medicine, University of Cologne, Cologne, Germany; 19 Division of Infectious Diseases, Klinikum Oldenburg, Oldenburg, Germany; 20 Mycotic Diseases Branch, Centers for Disease Control and Prevention, Atlanta, Georgia

**Keywords:** dematiaceous fungus, itraconazole, phaeohyphomycosis, voriconazole

## Abstract

**Background:**

Phaeohyphomycosis is infection caused by dematiaceous, or darkly pigmented, fungi. The spectrum of disease is broad, and optimal therapy remains poorly defined. The Mycoses Study Group established an international case registry of patients with proven/probable phaeohyphomycosis with the goal of improving the recognition and management of these infections.

**Methods:**

Patients from 18 sites in 3 countries were enrolled from 2009–2015. Cases were categorized as local superficial, local deep (pulmonary, sinus, osteoarticular infections), and disseminated infections. End points were clinical response (partial and complete) and all-cause mortality at 30 days and end of follow-up.

**Results:**

Of 99 patients, 32 had local superficial infection, 41 had local deep infection, and 26 had disseminated infection. The most common risk factors were corticosteroids, solid organ transplantation, malignancy, and diabetes. Cultures were positive in 98% of cases. All-cause mortality was 16% at 30 days and 33% at end of follow-up, and 18 of 26 (69%) with dissemination died. Itraconazole was most commonly used for local infections, and voriconazole was used for more severe infections, often in combination with terbinafine or amphotericin B.

**Conclusions:**

Phaeohyphomycosis is an increasingly recognized infection. Culture remains the most frequently used diagnostic method. Triazoles are currently the drugs of choice, often combined with other agents. Further studies are needed to develop optimal therapies for disseminated infections.

Phaeohyphomycosis refers to a group of infections caused by dematiaceous, or darkly pigmented, fungi from a variety of genera [1]. Although uncommon, these infections are increasingly seen in a variety of clinical syndromes in both immunocompromised and normal hosts [2, 3]. Infections due to certain species, such as *Lomentospora* (formerly *Scedosporium) prolificans*, have a very high mortality rate in immunocompromised patients despite aggressive therapy [4–6]. Other species (*Cladophialophora bantiana*, *Rhinocladiella mackenziei*) have been associated with brain abscesses in apparently normal individuals with similarly high mortality [2].

Dematiaceous fungi are generally found in soil or associated with plants and are distributed worldwide. Surveys of outdoor air for fungal spores routinely detect these moulds [7]. Some species appear to be geographically restricted, such as *R. mackenziei*, which is primarily seen in patients from the Middle East [8]. Dematiaceous fungi are difficult to classify into a simple framework of pathophysiology because they represent such a diverse group of organisms [9].

Optimal therapy for these infections remains uncertain. Data from the literature linking treatment and outcomes are sparse, with outcomes generally not well documented. Because these infections are so uncommon and varied in the fungal genera that cause disease, it is very unlikely that a prospective randomized trial will ever be performed. Therefore, the Mycoses Study Group established an international case registry of patients with phaeohyphomycosis in order to better define the epidemiology, treatment practices, and patient outcomes of these infections.

## METHODS

### Patients

Patients with proven/probable phaeohyphomycosis were enrolled from 18 participating sites in the United States, South America (Peru), and Australia. Cases were enrolled prospectively from January 1, 2009, through December 31, 2015. Criteria for study inclusion included: (1) culture or detection by polymerase chain reaction (PCR; performed at individual MSG site discretion) of a dematiaceous fungus from a normally sterile site (or bronchoalveolar lavage) or (2) histopathology confirming the presence of pigmented fungi in tissue specimens and (3) compatible clinical syndrome. This study was approved by each site’s institutional review board.

### Study Design

Cases were classified as *local superficial infections* involving only skin and subcutaneous tissues; *local deep infections* that included all other localized infections, such as infection of the sinuses, eyes, lungs, bones/joints, and other deep tissues; and *disseminated infections* that included fungemia, central nervous system (CNS) involvement, and disease at ≥2 noncontiguous sites. Patients with allergic sino-pulmonary disease were excluded. Chromoblastomycosis and mycetoma due to dematiaceous fungi were also included in this study. Although the genus *Scedosporium* includes many species, only *S. prolificans* was considered dematiaceous for the purposes of this study. *S. prolificans* is now placed in a new genus, *Lomentospora*, and will be referred to as *L. prolificans* for the remainder of this article [4].

Clinical data were entered into the Fungiscope database, an international global rare fungal infection registry (Fungiscope, www.fungiscope.net;www.clinicalsurveys.net). Data included demographic information, sites of disease, methods of diagnosis, clinical signs and symptoms, underlying diseases and predisposing factors, antifungal and/or surgical therapy, and clinical outcomes. Primary outcome measures were all-cause mortality and clinical response at 30 days post–diagnosis of phaeohyphomycosis. *Complete response* was defined as complete resolution of clinical and radiological findings, *partial response* was defined as improvement in clinical and radiological findings, and *failure* was evidence of stable disease or progression of infection. Secondary outcome measures were all-cause mortality and clinical response at end of the follow-up period for each patient.

### Mycology

Twenty-seven isolates were collected when available and sent to one of the investigators (SGR) for susceptibility testing using Clinical and Laboratory Standards Institute (CLSI) M38-A2 methodology [10]. These study isolates were also provided to the Centers for Disease Control (CDC) Fungal Reference Laboratory (Atlanta, Georgia), where sequencing of the internal transcribed spacer (ITS) gene of isolates confirmed species identification as previously described [11].

### Statistical Analysis

Statistical analysis included descriptive statistics (frequencies, means, medians) calculated for clinical, laboratory, and demographic variables. Chi-square analysis was used to compare groups.

## RESULTS

### Demographics

A total of 99 patients (91 proven cases and 8 probable cases) with unique infections and complete treatment and outcome data were enrolled; there were 59 men and 40 women. The mean age at diagnosis was 59 ± 15 years (range, 24–92 years) (Table 1). A total of 62 cases were from the United States, 30 from Australia, and 7 from Peru. In the United States, 19 cases were located in the South, 23 in the Midwest, 13 in the East, and 7 in the West.

**Table 1.** Demographics and Risk Factors for 99 Patients With Phaeohyphomycosis

	No. (%)			
	Total	Local- Superficial	Local-Deep	Disseminated
Demographics	(n = 99)	(n = 32)	(n = 41)	(n = 26)
Men	59 (60)	22 (69)	23 (56)	14 (54)
Women	40 (40)	10 (31)	18 (44)	12 (46)
Age, mean, y	59	57	61	57
Range	24–92	32–78	26–92	24–87
Ethnicity				
Caucasian	67 (68)	18 (56)	29 (71)	20 (77)
Hispanic	14 (14)	9 (28)	4 (10)	1 (4)
African	7 (7)	1 (3)	4 (10)	2 (8)
Asian	8 (8)	4 (13)	2 (5)	2 (8)
Arabic	3 (3)	0 (0)	2 (5)	1 (4)
Risk factor				
Stem cell transplantation	10 (10)	1 (3)	5 (12)	4 (15)
Graft vs host disease	6 (6)	0 (0)	4 (10)	2 (8)
Solid organ transplantation	33 (33)	17 (53)	9 (22)	7 (27)
Heart	2 (2)	1 (3)	1 (2)	0
Lung	9 (9)	4 (12)	5 (12)	0
Liver	2 (2)	1 (3)	1 (2)	0
Kidney	18 (18)	10 (31)	3 (7)	5 (19)
Pancreas	1 (1)	1 (3)	0	0
Intestine	1 (1)	0 (0)	0	1 (4)
HIV/AIDS	2 (2)	0 (0)	1 (2)	1 (4)
Chemotherapy	22 (22)	1 (3)	11 (27)	10 (38)
Neutropenia	22 (22)	1 (3)	10 (23)	11 (42)
Corticosteroid use	43 (43)	13 (41)	17 (41)	13 (50)
Malignancy	27 (27)	1 (3)	14 (34)	12 (46)
Other immunosuppression	16 (16)	2 (6)	8 (20)	6 (23)
Burn	1 (1)	1 (3)	0 (0)	0
Trauma	10 (10)	3 (9)	6 (15)	1 (4)
Diabetes mellitus	24 (24)	12 (38)	8 (20)	4 (15)
Alcohol abuse	3 (3)	0 (0)	2 (5)	1 (4)
Chronic liver disease	4 (4)	2 (6)	2 (5)	0
Chronic renal disease	7 (7)	3 (9)	2 (5)	2 (8)
Chronic pulmonary disease	13 (13)	4 (12)	8 (20)	1 (4)
No risk factor	10 (10)	5 (16)	3 (7)	2 (8)

### Risk Factors

The most common predisposing factors/comorbidities for infection with dematiaceous fungi were corticosteroid use (43%) and solid organ transplant (33%), and these were seen across all categories of infection (Table 1). Diabetes mellitus (24%) was often seen in patients with local superficial infections. In contrast, malignancy (27%), chemotherapy (22%), and neutropenia (22%) were frequent risk factors in patients with local deep and disseminated infections. No obvious risk factor was found in 10% of cases.

### Mycology

Certain fungal genera and species were more likely to cause specific syndromes (Table 2). *Alternaria*, *Exophiala*, and *Fonsecaea* were commonly associated with local superficial infections. Local deep infections were caused by 16 different genera, although only 3 (*Alternaria*, *Curvularia*, *Lomentospora*) were responsible for over half of these cases (53%). Almost all disseminated infections were caused by *Lomentospora*, *Cladophialophora*, and *Verruconis*. The most common pathogen was *L. prolificans*, isolated in 20% of all cases.

**Table 2.** Fungal Genera Isolated From 99 Patients With Phaeohyphomycosis

Genus	No. (%)			
	Total	Local- Superficial	Local-Deep	Disseminated
	(n = 99)	(n = 32)	(n = 41)	(n = 26)
*Acrophialophora*	1 (1)		1 (2)	
*Alternaria*	12 (12)	6 (17)	6 (14)	
*Aureobasidium*	1 (1)		1 (2)	
*Biatriospora*	1 (1)	1 (3)		
*Chaetomium*	3 (3)	1 (3)	2 (5)	
*Cladophialophora*	5 (5)			5 (18)
*Cladosporium*	2 (2)		2 (5)	
*Colletotricum*	1 (1)		1 (2)	
*Curvularia*	11 (11)	1 (3)	9 (22)	1 (4)
*Exophiala*	12 (12)	6 (17)	5 (12)	1 (4)
*Exserohilum*	4 (4)	2 (6)	2 (5)	
*Fonsecaea*	8 (8)	7 (22)		1 (4)
*Lomentospora*	20 (20)		8 (19)	12 (43)
*Madurella*	1 (1)	1 (3)		
*Medicopsis*	2 (2)	2 (6)		
*Microsphaeropsis*	2 (2)	1 (3)		1 (4)
*Paraconiothyrium*	1 (1)		1 (2)	
*Phaeoacremonium*	3 (3)	2 (6)	1 (2)	
*Phialophora*	2 (2)	2 (6)		
*Pleurostomaphora*	1 (1)		1 (2)	
*Scopulariopsis*	1 (1)		1 (2)	
*Ulocladium*	1 (1)		1 (2)	
*Verruconis*	5 (5)			5 (18)

Diagnosis was confirmed by culture in 97 of 99 (98%) cases. Using ITS sequencing, the CDC further identified 5 isolates to the species level, 2 unknown isolates were identified, and 1 isolate initially identified as *Phialophora verrucosa* was determined to be *Pleurostomophora richardsiae.* Histopathology showed granulomatous inflammation and/or fungal elements in 49 of 99 (49%) cases. PCR of tissue was positive in only 12 cases, 2 of whom had organisms noted on histopathological examination but culture showed no growth.

Results of antifungal susceptibility testing performed on 16 available isolates (11 isolates either became nonviable or did not sporulate) showed that extended spectrum azoles and terbinafine were generally the most active antifungal agents (Table 3). Other antifungal classes demonstrated limited activity, and *L. prolificans* and *S. brevicaulis* appeared to be resistant to all drugs.

**Table 3.** Minimum Inhibitory Concentration (µg/mL) for 16 Dematiaceous Fungal Isolates

Organism	AmB	5-FC	Itra	Posa	Vori	Isavu	Caspo	Mica	Anid	Terb
*Alternaria* spp.	0.5	>64	0.5	≤0.03	1	2	2	0.5	4	1
*Cladophialophora bantiana*	8	0.25	0.25	0.125	0.125	0.125	4	4	4	0.03
*Curvularia lunata*	0.25	>64	1	0.125	4	1	8	8	4	0.06
*Curvularia spicifera*	1	>64	0.5	0.125	1	8	1	2	8	0.125
*Exophiala* spp.	2	1	0.25	0.06	0.06	0.5	2	8	4	<0.03
*Exophiala oligosperma*	1	0.25	0.125	≤0.03	0.5	2	4	4	2	0.125
*Fonsecaea pedrosoi*	8	32	0.5	0.25	0.125	0.125	8	>16	16	<0.03
*Fonsecaea pedrosoi* (#2)	8	4	0.5	0.5	0.5	0.5	2	>16	4	<0.03
*Lomentospora prolificans*	>16	>64	>16	>16	>16	>16	16	>16	8	16
*Lomentospora prolificans* (#2)	>16	>64	>16	>16	>16	>16	16	>16	8	8
*Lomentospora prolificans* (#3)	16	>64	>16	>16	16	>16	16	>16	4	4
*Microsphaeropsis arundinis*	0.5	32	≤0.03	≤0.03	0.125	0.06	8	8	>16	0.25
*Phaeoacremonium sphinctophorum*	1	8	4	0.5	0.5	2	16	>16	4	0.25
*Pleurostomophora richardsiae*	4	4	1	0.5	0.5	0.5	4	>16	1	0.125
*Scopulariopsis brevicaulis*	16	>64	>16	>16	>16	>16	8	2	2	0.5
*Verruconis gallopava*	2	2	0.5	0.25	1	16	1	<0.03	<0.03	<0.03

Abbreviations: 5-FC, flucytosine; AmB, amphotericin B; anid, anidulafungin; caspo, caspofungin; isavu, isavuconazole; itra, itraconazole; mica, micafungin; posa, posaconazole; terb, terbinafine; vori, voriconazole.

### Clinical Manifestations and Laboratory Studies

#### Local Superficial Infections

Among the 32 patients who had local superficial infections, solid organ transplantation was the most common risk factor, occurring in 53% of patients, followed by corticosteroid use (41%) and diabetes mellitus (38%). All patients had skin and soft tissue infections, consisting of a variety of papules, plaques, nodules, and subcutaneous masses. There were 5 cases of chromoblastomycosis caused by *F. pedrosoi* and 2 cases of mycetoma; all of these were from Peru. The lesions were evenly distributed between upper (14) and lower (15) extremities, and there were 2 patients with lesions on the face and 1 with a lesion on the trunk. This pattern of distribution suggests direct traumatic inoculation as the most common mechanism of infection. Fever was present in only 5 patients.

All 32 of these patients were diagnosed by culture of the lesions, with 6 isolates having PCR performed to confirm the species identification. Histopathology was reported for 23 cases. In 21 cases, hyphae or fungal elements were seen, and in 8 of these granulomas also were noted. One specimen had granulomas only, and 1 had neither fungal elements nor granulomas seen.

#### Local Deep Infections

The local deep infection group was comprised of 41 patients who had a variety of clinical syndromes. The most common sites of involvement were pulmonary (13 cases), sinuses (11 cases), and bone/joint (10 cases). Fever was present in 15 of 41 (37%) patients. Twelve of the 13 patients (92%) who had pulmonary infection had dyspnea, 10 (77%) had cough, and 2 (15%) had pleuritic chest pain; none had hemoptysis. For those patients who had invasive sinusitis, 7 (64%) had sinus pain and 6 (55%) had facial swelling; only 3 had an eschar seen on sinus endoscopy.

The most common genera causing local deep infection were *Alternaria*, *Curvularia*, and *Lomentospora.* Diagnosis was made by culture in all but one of the 41 patients; in that patient, PCR of sinus tissue revealed *Colletotricum gleosporioides*. Of the 17 biopsies that were performed, 15 had fungal hyphae noted and 3 showed granulomatous inflammation.

#### Disseminated Infections

Almost all of the 26 patients in the disseminated group had some risk factor identified. The 2 patients who had no risk factors identified had brain abscesses due to *C. bantiana*. CNS involvement was present in 12 patients, pulmonary infection in 12, and endocarditis in 4. Fever, observed in 14 (54%) patients, was significantly more frequent than in patients with local infection (54% vs 27%; *P* = .01). Nodular rash was observed in 5 patients and neurologic symptoms in 9 patients. The organism most commonly causing disseminated infection was *L. prolificans*, with *C. bantiana* and *Verruconis gallopava* being the next most common.

The 4 cases of endocarditis occurred in women; different species were found in each: *L. prolificans*, *V. gallopava*, *F. pedrosoi*, and *Curvularia lunata.* Two of these patients were solid organ transplant recipients and were receiving corticosteroids, but the other 2 were not immunosuppressed. Blood cultures were positive in all cases except for the patient who had *F. pedrosoi* endocarditis, who had an echocardiogram showing a mobile left ventricular vegetation and grew the organism from biopsy of a skin lesion. All patients had large (>1-cm) vegetations noted on echocardiography.

Primary brain abscess occurred in 6 patients (4 men and 2 women); 5 were caused by *C. bantiana* and 1 by *V. gallopava*. One patient had received a stem cell transplant, and 3 had received corticosteroids prior to diagnosis. The other 2 patients had no risk factors identified.

Culture was positive in 25 of the 26 patients; 1 patient had a diagnosis of *C. bantiana* infection established only by PCR of brain tissue. Blood cultures yielded a mold in 13 patients, 12 of whom had *L. prolificans* and 1 had *V. gallopava*. Of 13 patients who had a biopsy performed, histopathology revealed fungal elements in 11 and granulomas in 2.

### Treatment and Outcomes

#### Local Superficial Infections

Treatment of the 32 patients who had local superficial infections was primarily with antifungal drugs (Table 4). In addition, 6 patients had surgical intervention and 5 underwent cryotherapy, the latter for chromoblastomycosis. One patient was considered cured with excision of a skin nodule without antifungal therapy. Antifungal regimens varied and were often prolonged; median treatment duration was 73 days (range, 1–915 days). Itraconazole was given to 13 patients, voriconazole to 9, and posaconazole to 10. Ten patients received several drugs sequentially, and 2 received concomitant itraconazole and terbinafine.

**Table 4.** Therapy for Phaeohyphomycosis in 99 Patients

Therapy^a^	No. (%)			
	Total	Local- Superficial	Local-Deep	Disseminated
	(n = 99)	(n = 32)	(n = 41)	(n = 26)
Monotherapy				
Lipid AmB	15 (15)	4 (13)	9 (22)	2 (8)
Itraconazole	21 (16)	13 (41)	7 (17)	1 (4)
Voriconazole	32 (32)	9 (28)	14 (34)	9 (35)
Posaconazole	16 (16)	10 (31)	5 (12)	1 (4)
Isavuconazole	2 (2)	1 (3)	1 (2)	0 (0)
Fluconazole	1 (1)	0 (0)	1 (2)	0 (0)
Caspofungin	2 (2)	1 (3)	0 (0)	1 (4)
Micafungin	2 (2)	1 (3)	1 (2)	0 (0)
Terbinafine	1 (1)	1 (3)	0 (0)	0 (0)
Combination therapy				
Azole + terbinafine	12 (12)	2 (6)	4 (10)	6 (23)
Azole + AmB	14 (14)	1 (3)	9 (22)	4 (15)
Azole + echinocandin	2 (2)	1 (3)	1 (2)	0 (0)
Azole + 5-FC	1 (1)	0 (0)	0 (0)	1 (4)
AmB + echinocandin	1 (1)	0 (0)	1 (3)	0 (0)
Triple-drug combination	6 (6)	0 (0)	1 (2)	5 (19)
Four-drug combination	1 (1)	0 (0)	0 (0)	1 (4)
Surgery	33 (33)	6 (19)	20 (49)	7 (27)
Cryotherapy	5 (5)	5 (12)	0 (0)	0 (0)

Abbreviations: 5-FC, flucytosine; AmB, amphotericin B; Azole, itraconazole, voriconazole, or posaconazole

^a^Some patients received more than 1 antifungal or combinations of antifungal agents at different times.

The primary outcomes of clinical response and mortality at 30 days were 79% and 3%, respectively (Table 5). By day 30, most had only a partial response. The clinical response at end of follow-up was 84%, with 11 patients (34%) showing a partial response and 16 (50%) having a complete response. Two patients died, but neither death was related to the fungal infection.

**Table 5.** Outcomes of 99 Patients With Phaeohyphomycoses

Outcome	No. (%)		
	Local-Superficial	Local-Deep	Disseminated
	(n = 32)	(n = 41)	(n = 26)
30-day response			
Complete	4 (13)	5 (12)	2 (8)
Partial	21 (66)	17 (41)	6 (23)
Failure	7 (22)	19 (46)	18 (69)
End-of-follow-up response			
Complete	16 (50)	18 (44)	3 (12)
Partial	11 (34)	10 (24)	5 (19)
Failure	5 (16)	13 (32)	18 (69)
Mortality			
30 d	1 (3)	5 (12)	10 (38)
End of follow-up	2 (6)	13 (32)	18 (69)
Due to fungal infection	0 (0)	4 (10)	13 (50)
Follow-up, median (range), d	189 (14–1006)	130 (2–1155)	69 (1–1104)

#### Local Deep Infections

Therapy included surgery in 20 of the 41 cases. In only 1 case (tenosynovitis) was surgery alone curative; all other patients received concomitant antifungal therapy. The median duration of antifungal treatment was 50 days (range, 3–710 days). Voriconazole was used in 26 of 40 (65%) patients, 12 of whom received this agent in combination with other antifungal drugs, usually lipid formulation amphotericin B.

The primary outcomes of clinical response and mortality at 30 days were 53% and 12%, respectively. At the end of follow-up, the clinical response rate was 68%, with 18 patients (44%) having a complete response and 10 (24%), a partial response. These rates did not differ between those receiving combination therapy vs monotherapy. At end of follow-up, 13 patients had died; 4 deaths were attributed to fungal infection.

#### Disseminated Infections

Sixteen of the 26 patients who had disseminated infection were treated with combination antifungal therapy and 10 with monotherapy. The median duration of drug treatment was 61 days (range, 2–720 days). Ten patients received 2-drug therapy, 5 patients received 3-drug therapy, and 1 patient was given 4 drugs (L-AmB + voriconazole + flucytosine + micafungin) concurrently.

Among the endocarditis cases, only the patient who had *C. lunata* infection underwent valve replacement. This patient also received posaconazole + terbinafine and was alive at 5 months. The other surviving patient, who had *V. gallopava* endocarditis, received voriconazole + terbinafine + anidulafungin for 1 year. The remaining 2 patients received monotherapy with voriconazole, and both died. All 6 patients with brain abscess received combination therapy with L-AmB + voriconazole or posaconazole. Additionally, 1 patient also received 5-FC, 1 terbinafine, and 1 5-FC and micafungin. Four patients underwent surgery. Only 2 of the 6 patients were alive at 3 months.

In disseminated cases, primary outcomes of clinical response and mortality at 30 days were 31% and 38%, respectively. At end of follow-up, clinical response was seen in 5 of 16 (31%) patients who were treated with combination therapy vs 3 of 10 (30%) patients who had received monotherapy. Eighteen patients (69%) died, with 13 deaths attributed to fungal infection. Eleven (69%) patients receiving combination therapy died compared with 7 (70%) receiving monotherapy. Patients with *L. prolificans* infection had mortality of 100% compared with 36% for infection due to other fungi (*P* < .01), which may reflect the impact of underlying disease. At end of follow-up, those with disseminated infection compared with those with local deep infections had significantly worse clinical response, 31% vs 68% (*P* < .01), and mortality, 69% vs 32% (*P* < .01).

## DISCUSSION

Dematiaceous fungi are increasingly important causes of disease [9]. Numerous species have been implicated in a variety of different clinical syndromes [9]. This diversity presents a challenge to efforts to develop useful, consistent guidelines for management [12]. The literature contains mostly anecdotal clinical reports that are subject to publication bias, making it difficult to derive more general recommendations regarding management of these infections. This study represents a comprehensive attempt to formally study and provide a frame of reference for these heterogeneous and often refractory infections.

The pathogenesis of infections due to dematiaceous fungi is not well understood. Superficial infections are generally considered secondary to local trauma and manifest little tissue invasion; disseminated infection is uncommon except in immunocompromised individuals. A likely candidate virulence factor for these organisms is melanin, which is present in the cell wall of all dematiaceous fungi. In experimental animal models, disruption of specific genes involved in melanin production leads to markedly reduced virulence [13, 14]. There are several mechanisms proposed by which melanin may act as a virulence factor, including scavenging free radicals and hypochlorite produced by phagocytic cells in the oxidative burst, thus preventing killing of the organisms [15]. Recently, mutations in a specific host gene (CARD9) have been associated with an increased risk of developing disseminated infection with certain dematiaceous fungi [16]. Additional studies are needed to further elucidate genetic susceptibility to these many disparate fungi.

The diagnosis of phaeohyphomycosis, as noted in our study, primarily relies on careful examination of growth that appears in culture and histopathological examination of tissue specimens. Not only the routine hematoxylin and eosin stain, but also the Fontana-Masson stain, which is specific to melanin, should be used [17]. There are no specific non-culture-based methods that are routinely available to aid in the diagnosis of phaeohyphomycosis. Available antigen-based tests, such as galactomannan and (1,3)-β-D-glucan assays, have not proved useful and were rarely utilized in our registry [18, 19]. PCR of highly conserved regions of ribosomal DNA has the potential to be a useful technique for identification of dematiaceous organisms [20], and ITS and D1/D2 analyses are becoming more available for identification of this diverse group of fungi from clinical specimens [21]. Matrix Assisted Laser Desorption/Ionization Time of Flight (MALDI-ToF) mass spectroscopy may also become an accurate method for dematiaceous mold identification as reference databases for this technique improve [22, 23].

The European Confederation of Medical Mycology, together with the European Society of Clinical Microbiology and Infectious Diseases, recently published recommendations for therapy of phaeohyphomycosis [12]. These recommendations reflect the challenges encountered because of the lack of randomized controlled trial data for these fungal infections. The guidelines suggest itraconazole, voriconazole, and posaconazole as active drugs, with voriconazole preferred for CNS infection and posaconazole utilized for salvage therapy [12]. In our registry, voriconazole was the most commonly used agent, primarily because of its in vitro activity, better oral absorption and tolerability, and the accumulated experience with this agent for other types of invasive mold infections. Itraconazole is still widely used in developing countries, and posaconazole occasionally is used for refractory infections. There are limited in vitro data and clinical experience for the use of isavuconazole for treating phaeohyphomycoses [24, 25]. In our study, isavuconazole was used only twice as salvage therapy, with 1 partial response and 1 failure. Interestingly, duration of therapy was longer in local superficial infections than local deep or disseminated disease; this finding is possibly due to earlier deaths in patients with severe disease.

The guidelines suggest using combination antifungal therapy for CNS and disseminated infection [12], and combination therapy is often used for more refractory infections [26, 27]. Voriconazole or posaconazole with terbinafine have been used in selected cases of *L. prolificans* infection based on in vitro data showing synergistic activity of terbinafine with these azoles and promising, but limited, clinical data. However, we found no improvement in outcomes with combination therapy, but acknowledge that there were small numbers of patients with these more severe infections entered into the registry. Given the benign nature of using an extended spectrum triazole with terbinafine or an echinocandin, continued use of these agents in combination is likely, especially for infection with *L. prolificans*, which generally is resistant in vitro to all antifungals [6].

We have collected a very large database of well-documented cases of phaeohyphomycosis derived from diverse clinical sites. Our hope is that this report is helpful to clinicians and may spur additional efforts to systematically study these uncommon, often refractory infections. Therapy will undoubtedly continue to evolve and improve as additional clinical data accumulate regarding management of specific clinical syndromes.
